# Boys born with hypospadias and fetal growth restriction exhibit shorter anogenital distances: a retrospective cross-sectional study

**DOI:** 10.3389/fped.2025.1602368

**Published:** 2025-07-09

**Authors:** Wenfeng Pan, Min Wu, Yan Chen, Hua Xie, Yichen Huang, Fang Chen

**Affiliations:** Department of Urology, Shanghai Children’s Hospital, School of Medicine, Shanghai Jiao Tong University, Shanghai, China

**Keywords:** hypospadias, anogenital distances, fetal growth restriction, low birth weight (LBW), small for gestational age (SGA)

## Abstract

**Background:**

Hypospadias is a common congenital urological malformation in males, potentially associated with inadequate prenatal androgen exposure. Anogenital distances (AGDs) are biomarkers of prenatal androgen action, while fetal growth restriction (FGR) may impair gonadal development and hormone levels. This study aims to investigate the relationship between AGDs and different severities of hypospadias, with a specific focus on the impact of FGR.

**Methods:**

A retrospective observational study was conducted on male pediatric patients treated at Shanghai Children's Hospital between August 2019 and January 2023. Patients were divided into the control group and the hypospadias group, with the latter further classified into distal, middle, and proximal subgroups based on urethral meatus location. AGDs, including anoscrotal distance (ASD), AGD-1, and AGD-2, were measured under anesthesia. Linear regression analysis was performed to assess the associations between AGDs, hypospadias severity, and FGR indicators, including low birth weight (LBW) and small for gestational age (SGA).

**Results:**

A total of 386 pediatric patients were included, with 205 in the control group and 181 in the hypospadias group. Patients with hypospadias exhibited significantly shorter AGDs compared to the control group (*P* < 0.05). Among hypospadias subtypes, AGDs showed a decreasing trend with increasing severity of hypospadias (e.g., ASD: 39.0 ± 12.8 mm in distal vs. 31.8 ± 8.6 mm in proximal cases, *P* < 0.05). Linear regression analysis revealed that proximal hypospadias and SGA were significantly associated with shorter AGDs across all measurements (e.g., proximal hypospadias reduced ASD by 6.52 mm, 95% CI: −9.97 to −3.06, *P* < 0.001; SGA reduced ASD by 4.48 mm, 95% CI: −8.00 to −0.97, *P* = 0.01). Prematurity showed no significant association with AGDs.

**Conclusion:**

Boys with hypospadias and FGR exhibit significantly shorter AGDs, with more severe hypospadias and SGA showing the strongest associations. This study provides a foundation for future clinical assessments and research into prenatal factors influencing male genital development.

## Introduction

Hypospadias is one of the most common congenital urological malformations, with a global incidence rate estimated at 20.9 per 10,000 live births (1). Recent epidemiological studies have demonstrated associations between hypospadias and factors such as preterm birth, low birth weight (LBW), and being small for gestational age (SGA) (2–4). These factors are hypothesized to be linked to fetal growth restriction (FGR), which refers to the situation where the fetal growth rate fails to reach the full growth potential conferred by the fetus's genes and may play a critical role in the development of hypospadias. Evidence suggests that FGR can impair placental function, leading to insufficient production of human chorionic gonadotropin and may result in reduced testosterone secretion in the fetus (5, 6). Maternal androgens can regulate the process of placental angiogenesis and an intrauterine hyperandrogenic environment has adverse effects on the proliferation, decidualization and material transportation functions of the endometrium. This results in inadequate prenatal androgen exposure, potentially contributing to the development of hypospadias (7).

Anogenital distances (AGDs) serve as key biomarkers of prenatal androgen action and exposure, particularly in rodent models (8). Clinical studies have consistently shown that shortened AGDs are associated with hypospadias, cryptorchidism, and impaired male fertility (9–11). However, it remains unclear whether AGDs are altered in children with hypospadias and concurrent FGR. To date, no studies have specifically addressed this question.

Therefore, this study aims to investigate the relationship between AGDs and different subtypes of hypospadias in pediatric patients, with a particular focus on those with FGR. By identifying potential associations, this research seeks to provide a foundation for future clinical assessments and improve the understanding of prenatal androgen exposure as a determinant of urogenital development.

## Methods and materials

### Study design

This study is a single-center retrospective observational study conducted in accordance with the principles of the Declaration of Helsinki. Ethical approval was obtained from the Ethics Committee of Shanghai Children's Hospital (No. 2014R022-E06). The study included pediatric patients treated at Shanghai Children's Hospital between August 2019 and January 2023. The study period was selected to ensure sufficient sample size and data completeness. Data were sourced from a prospective database, medical records, and AGD measurement data. This study follows the Strengthening the Reporting of Observational Studies in Epidemiology (STROBE) guidelines.

Patients were divided into two groups: the control group and the hypospadias group. The control group included children with redundant prepuce or phimosis who underwent circumcision. Patients with concealed penis, micropenis, or other congenital malformations were excluded. The hypospadias group included children diagnosed with hypospadias, excluding those with chromosomal abnormalities, cryptorchidism, isolated penile curvature, or other associated congenital malformations.

### Data collection and group classification

Basic information such as age, height, weight, birth weight and gestational age, birth length of the pediatric patients was retrospectively collected. Physical examinations were conducted by the pediatric urologist, and the patients were subsequently classified into the control group and the hypospadias group. Specifically, for patients with hypospadias, they were further divided into three subgroups based on the location of the urethral meatus: distal hypospadias (urethral meatus located at the glans penis, coronal sulcus, or lower coronal sulcus), middle hypospadias (urethral meatus located on the penile shaft), and proximal hypospadias (urethral meatus located on the penoscrotal, scrotal, or perineal region) (12). Based on gestational age (GA), patients were classified into the full-term group (37 weeks ≤ GA ≤ 42 weeks) and the preterm group (28 weeks ≤ GA < 37 weeks) (13). According to BW, patients were categorized into the normal-birth-weight (NBW) group (BW ≥ 2,500 g) and the LBW group (BW < 2,500 g) (14). Finally, based on gestational age at birth, patients were classified into the appropriate for gestational age (AGA) group and the SGA group (15).

### Measurement of AGDs

**Figure 1:**
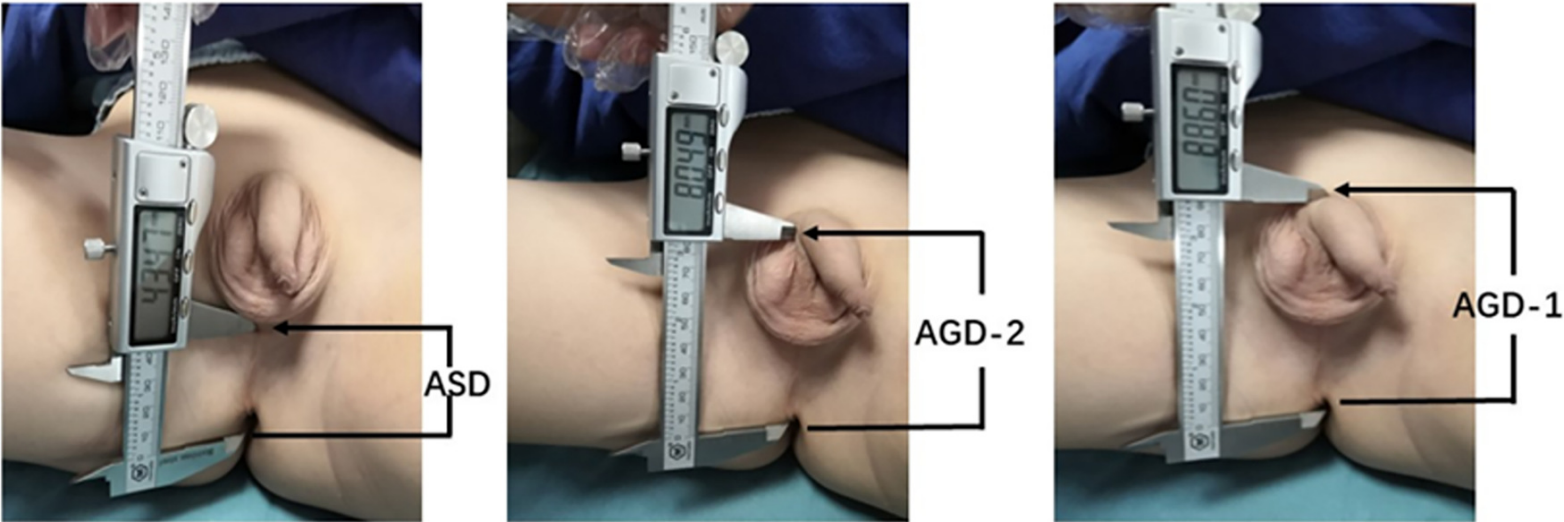
Measurement of anogenital distances (AGDs) in male children. ASD: anoscrotal distance, the distance from the midpoint of the anus to the base of the scrotum; AGD-2: anogenital distance-2, the distance from the midpoint of the anus to the ventral base of the penile base; AGD-1: anogenital distance-1, the distance from the midpoint of the anus to the dorsal base of the penile base.

The measurement of AGDs was conducted under anesthesia (Figure 1). The specific method for measuring AGDs involved positioning the pediatric patient in the supine position with their perineal area fully exposed (16). With the assistance of two individuals, the newborn's hips were flexed and abducted to create a “frog-leg” position. The pediatric surgeon then used a vernier caliper to measure the anogenital distances three times and calculated the average value. The measurement accuracy of AGDs is 0.01 mm. It included measuring the anoscrotal distance (ASD), which is the distance from the midpoint of the anus to the base of the scrotum, as well as AGD-2, which is the distance from the midpoint of the anus to the ventral aspect of the penile base, and AGD-1, which is the distance from the midpoint of the anus to the dorsal aspect of the penile base.

### Statistical methods

All data were statistically analyzed using SPSS 24.0 and R 4.3.2 software. The Kolmogorov–Smirnov test was employed to assess the normality of all data. For data that followed a normal distribution, the *t*-test was used for comparisons between groups. For data that did not follow a normal distribution, the Mann–Whitney test was utilized to analyze differences. To compare mean differences between groups, either Student's *t*-test with Bonferroni *post-hoc* testing or one-way analysis of variance (ANOVA) was applied. Linear regression analysis was employed to evaluate the relationship between AGDs and various factors, including hypospadias severity, SGA, LBW, and prematurity. Data are presented as mean ± standard deviation. A corrected *P*-value <0.05 was considered statistically significant.

## Results

### General information

**Figure 2:**
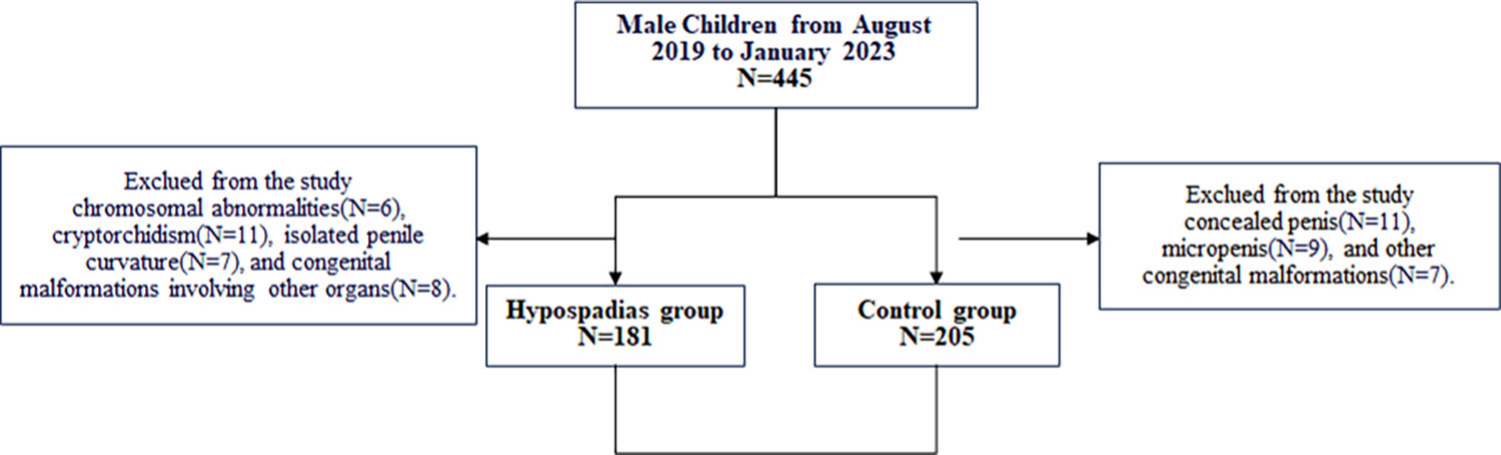
Flow chart of study participants.

A total of 386 pediatric patients were included in this study (Figure 2), with a mean age of 5.0 ± 1.0 years (range: 2.6–7.5 years). Patients were categorized into the control group (*n* = 205) and the hypospadias group (*n* = 181). The average age was 5.1 ± 1.1 years in the control group (range: 2.6–7.5 years) and 5.0 ± 0.8 years in the hypospadias group (range: 3.0–7.4 years). Significant differences were observed between the two groups regarding the incidence of preterm birth, low birth weight (LBW), and small for gestational age (SGA) (Table 1).

**Table 1:** Comparison of general characteristics between control group and hypospadias group.

General information		Control group (*N* = 205)	Hypospadias group (*N* = 181)	*P* value
Age (years)		5.1 ± 1.1	5.0 ± 0.8	0.63
Height (m)		1.1 ± 0.1	1.1 ± 0.1	0.64
Weight (kg)		21.0 ± 7.0	19.0 ± 12.6	0.06
Gestational age (w)		39.0 ± 1.62	38.2 ± 2.4	<0.001
Birth weight (g)		3,368.0 ± 431.06	2,919.8 ± 702.7	<0.001
Preterm, *n* (%)	Yes	7 (3.4)	33 (18.2)	<0.001
	No	198 (96.6)	148 (81.8)	
Birth weight, *n* (%)	<2,500 g	8 (3.9)	46 (25.4)	<0.001
	≥2,500 g	197 (96.1)	135 (74.6)	
Small for gestational age, *n* (%)	Yes	9 (4.4)	47 (26.0)	<0.001
	No	196 (95.6)	134 (74.0)	

### Comparison of AGDs between the control group and the hypospadias group

**Figure 3:**
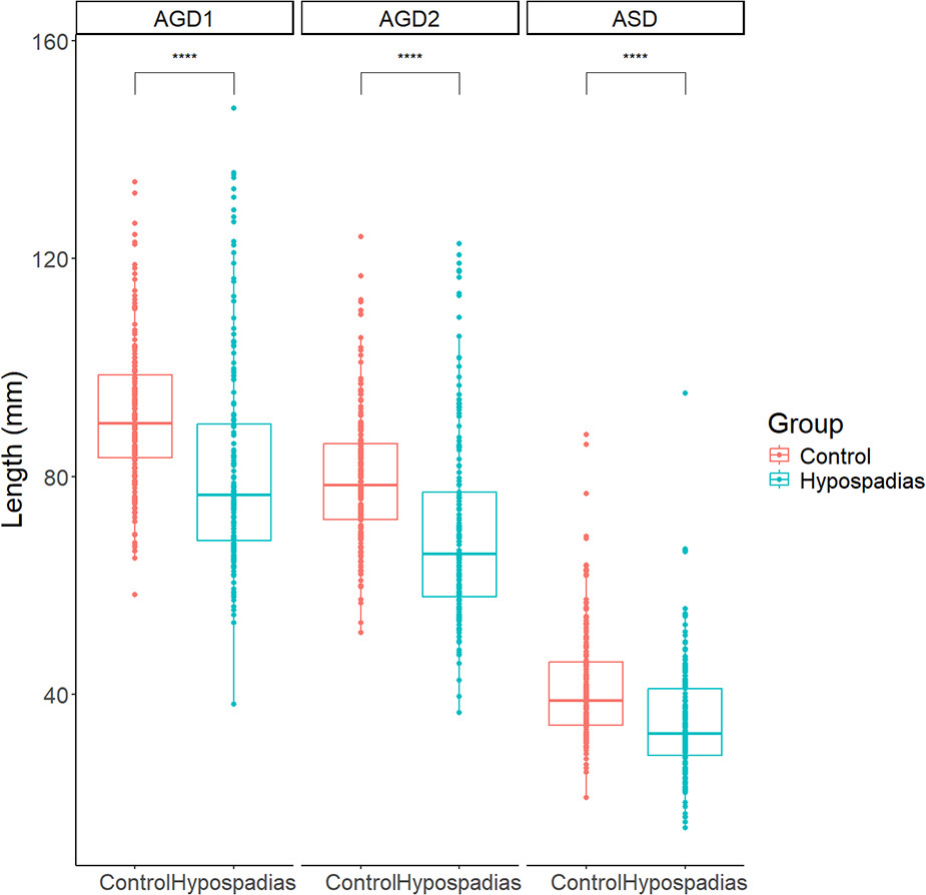
Summary chart of AGDs in control group and experimental group. ASD: anoscrotal distance, the distance from the midpoint of the anus to the base of the scrotum; AGD-2: anogenital distance-2, the distance from the midpoint of the anus to the ventral base of the penile base; AGD-1: anogenital distance-1, the distance from the midpoint of the anus to the dorsal base of the penile base. ***P < 0.001 indicated statistical significance.

No significant differences in age, height, or weight were observed between the control and hypospadias groups. However, children with hypospadias showed significantly shorter anogenital distances (AGDs) compared to the control group (Figure 3). Specifically, the mean ASD was 35.1 ± 10.4 mm in the hypospadias group, significantly shorter than 41.2 ± 10.1 mm in the control group (*P* < 0.05). Similarly, AGD2 and AGD1 were reduced in the hypospadias group (69.9 ± 17.4 and 81.8 ± 19.4 mm, respectively) compared to the control group (79.8 ± 11.9 and 91.2 ± 12.8 mm, *P* < 0.05 for both).

### Comparison of AGDs among different types of the hypospadias group

Among the children with hypospadias (Table 1), there were 58 cases of distal hypospadias (32.0%), 49 cases of middle hypospadias (27.1%), and 74 cases of proximal hypospadias (40.9%), respectively. Additionally, 148 children were full-term infants (81.8%), while 33 were premature infants (18.2%). Regarding BW, 135 children had normal birth weight (74.6%), and 46 children were LBW (25.4%). Furthermore, 134 children were AGA (74.0%), while 47 children were SGA (26.0%).

**Figure 4:**
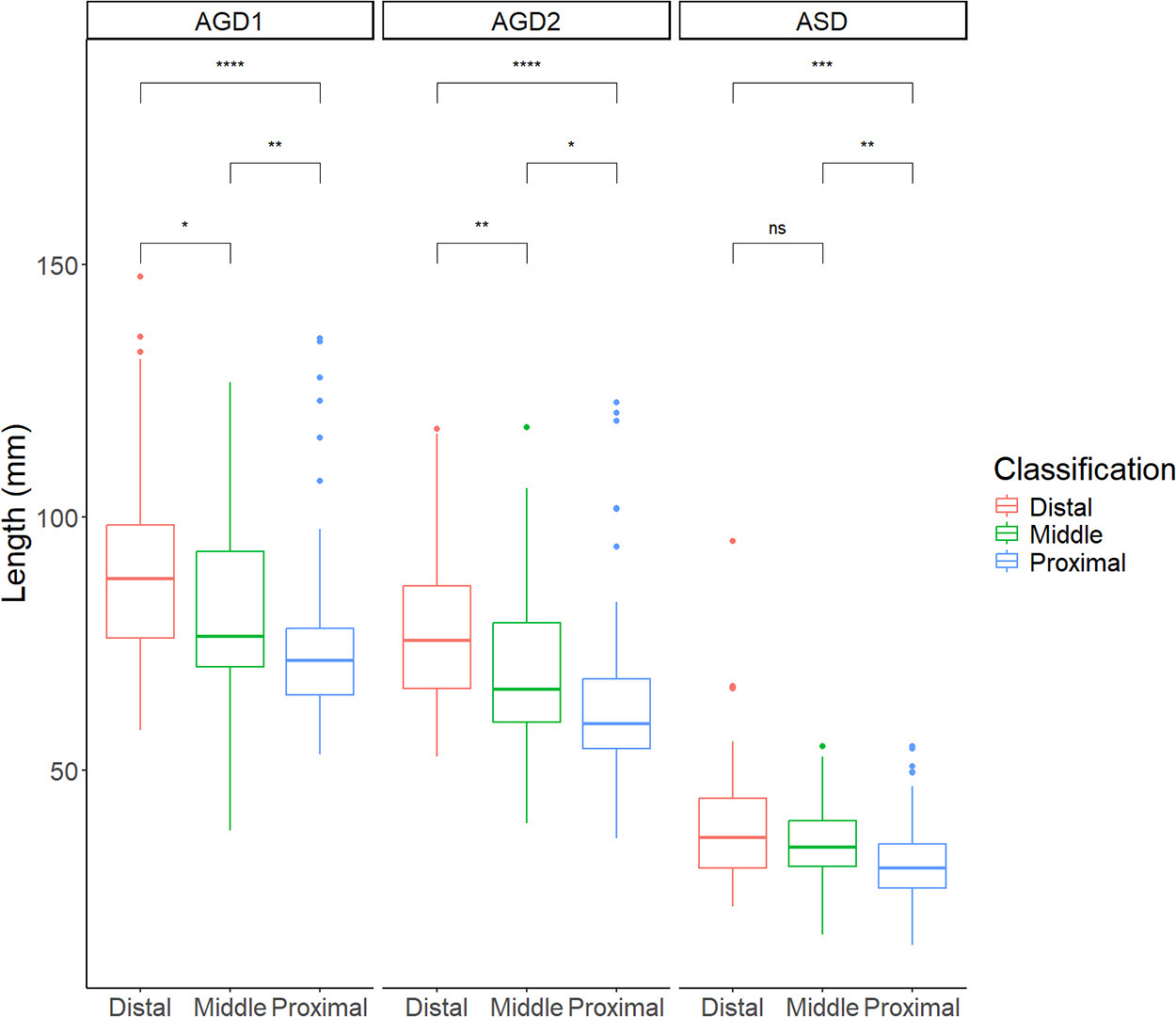
Summary chart of AGDs in children with hypospadias of different severities. ASD: anoscrotal distance, the distance from the midpoint of the anus to the base of the scrotum; AGD-2: anogenital distance-2, the distance from the midpoint of the anus to the ventral base of the penile base; AGD-1: anogenital distance-1, the distance from the midpoint of the anus to the dorsal base of the penile base. ***P < 0.001, **P < 0.01, *P < 0.05 and ^ns^*P* > 0.05 indicated statistical significance.

Significant differences in AGDs were observed among the distal, middle and proximal hypospadias groups, with shorter AGDs corresponding to increasing severity (Figure 4). The mean ASD was 39.0 ± 12.8 mm in the distal group, 35.4 ± 8.1 mm in the middle group, and 31.8 ± 8.6 mm in the proximal group. Similarly, AGD2 measurements decreased with severity, being 78.0 ± 15.7, 69.9 ± 16.9, and 63.5 ± 16.5 mm in the distal, middle and proximal groups, respectively. A similar trend was noted for AGD1, with values of 89.7 ± 19.4, 82.6 ± 19.1, and 75.0 ± 17.2 mm, respectively.

### Linear regression analysis of AGDs

The linear regression analysis demonstrated that proximal hypospadias and SGA were significantly associated with shorter AGDs across all measurements, while prematurity showed no significant relationship (Table 2, Supplementary Figure S1). Specifically, proximal hypospadias was associated with reductions of 13.43 mm in AGD1 (95% CI: −19.79 to −7.06, *P* < 0.001), 13.41 mm in AGD2 (95% CI: −19.09 to −7.73, *P* < 0.001), and 6.52 mm in ASD (95% CI: −9.97 to −3.06, *P* < 0.001). SGA was also linked to decreased AGD1 (Estimate: −8.04 mm, 95% CI: −14.53 to −1.56, *P* = 0.02), AGD2 (Estimate: −6.84 mm, 95% CI: −12.62 to −1.06, *P* = 0.02), and ASD (Estimate: −4.48 mm, 95% CI: −8.00 to −0.97, *P* = 0.01). Middle hypospadias only showed a significant reduction in AGD2 (Estimate: −7.01 mm, 95% CI: −13.27 to −0.75, *P* = 0.03), while prematurity had no significant effect on any AGD measurements. These findings suggest that the severity of hypospadias and fetal growth restriction (SGA) are critical factors associated with shorter AGDs, reflecting the potential role of prenatal growth and androgen exposure in urogenital development.

**Table 2:** Linear regression analysis of AGDs and different severities of hypospadias and SGA.

AGDs	Estimate	95% CI	Std. error	*t* value	*P* value
AGD1					
(Intercept)	91.15	86.23 to 96.06	2.49	36.60	<0.001
Middle hypospadias	−5.87	−12.89 to 1.15	3.56	−1.65	0.10
Proximal hypospadias	−13.43	−19.79 to −7.06	3.23	−4.16	<0.001
SGA	−8.04	−14.53 to −1.56	3.29	−2.45	0.02
Premature	−1.11	−8.37 to 6.15	3.68	−0.30	0.76
AGD2					
(Intercept)	79.09	74.71 to 83.47	2.22	35.62	<0.001
Middle hypospadias	−7.01	−13.27 to −0.75	3.17	−2.21	0.03
Proximal hypospadias	−13.41	−19.09 to −7.73	2.88	−4.66	<0.001
SGA	−6.84	−12.62 to −1.06	2.93	−2.34	0.02
Premature	−0.29	−6.76 to 6.19	2.28	−0.09	0.93
ASD					
(Intercept)	39.87	37.21 to 42.54	1.35	29.54	<0.001
Middle hypospadias	−2.93	−6.73 to 0.88	1.93	−1.52	0.13
Proximal hypospadias	−6.52	−9.97 to −3.06	1.75	−3.73	<0.001
SGA	−4.48	−8.00 to −0.97	1.78	−2.52	0.01
Premature	−0.74	−4.68 to 3.19	2.00	−0.37	0.71

SGA, small for gestational age; AGDs, anogenital distances; ASD, anoscrotal distance, the distance from the midpoint of the anus to the base of the scrotum; AGD-2, anogenital distance-2, the distance from the midpoint of the anus to the ventral base of the penile base; AGD-1, anogenital distance-1, the distance from the midpoint of the anus to the dorsal base of the penile base.

## Discussion

This study included 205 control children and 181 children with hypospadias, comparing the AGDs between normal healthy children and those with hypospadias, as well as among different types of hypospadias patients (preterm birth, LBW and SGA). The results showed that children with hypospadias and FGR had significantly shortened AGDs, potentially due to reduced prenatal androgen exposure resulting from placental dysfunction. This provides important insights for future clinical assessments and helps to better understand the pathogenesis of hypospadias.

Over the past three decades, the incidence of hypospadias has shown an upward trend year by year. This phenomenon may be related to genetic mutations and environmental factors, although there is no clear explanation (17). Studies have suggested that environmental factors may disrupt the intrauterine balance of androgens and estrogens, leading to an increase in hypospadias in offspring (18). The masculine programming window (MPW) is currently considered a critical period for reproductive system development, possibly occurring between weeks 8 and 14 of human pregnancy. During this period, exposure to endocrine disrupting chemicals in the pregnancy environment or decreased androgenization may lead to changes in AGDs and penile length, resulting in congenital urogenital malformations (19). This may be influenced by fetal, placental, or maternal factors, including FGR, which increases the risk of hypospadias in offspring (20). FGR is an important maternal-fetal factor that can cause placental insufficiency, resulting in weakened or inadequate prenatal androgen exposure during the MPW (5). After birth, it mainly manifests as LBW and SGA, with birth weights lower than the 10th percentile for the same gestational age (21). These infants are often born with multiple congenital urologic malformations, such as hypospadias, cryptorchidism, and micropenis (22).

AGDs, the distances from the midpoint of the anus to the genitalia, were first discovered by human reproductive toxicologists in rodent experiments and used to distinguish gender (23). Based on previous studies, the currently recognized male AGDs are ASD, AGD-2 and AGD-1 (9–11). Subsequent studies have shown that AGDs are not only important biomarkers in human reproductive toxicology but also useful for assessing exposure levels to environmental endocrine disruptors and as indicators of fetal androgen exposure levels (24). Some studies even suggest a possible link with individual genetic factors, androgen secretion disorders, or receptor defects (25, 26). They are also associated with reproductive health outcomes in humans, such as cryptorchidism, hypospadias, low fertility, and polycystic ovaries (9, 11, 26, 27).

Multiple scholars have found shorter AGDs in children with hypospadias, which may be related to androgen deficiency (26, 28). Based on these retrospective studies, Singal (10) and Cox (29) conducted prospective studies to explore the relationship between AGDs and the severity of hypospadias. They found that children with hypospadias had shorter AGDs, and those with proximal hypospadias had shorter AGDs than those with distal or middle hypospadias, suggesting more severe defects in intrauterine androgen action in children with proximal hypospadias. This is consistent with the conclusions of our study: AGDs were shorter in proximal hypospadias than in distal hypospadias, showing a negative association. As most studies have focused on non-Asian populations, there are few studies on the relationship between AGDs and hypospadias in Asian populations, and this study addresses this gap.

Studies have shown that FGR may also affect the occurrence of hypospadias in offspring, including fetal, placental function, and maternal factors. Previous epidemiological studies have found a close relationship between LBW and hypospadias, but the underlying mechanism of this association is unclear (30). Meanwhile, studies have also found that children with proximal hypospadias often have low birth weights, especially among SGA infants, where the proportion of hypospadias is significantly increased (31, 32). Hsieh found in preterm birth infants with hypospadias that their initial birth weights were low, but they exhibited a catch-up growth pattern at 1 year of age (28). This may be related to FGR or placental dysfunction, leading to reduced human chorionic gonadotrophin secretion by the placenta, resulting in abnormal testosterone secretion required for fetal genital masculinization, and thus reduced prenatal masculinization levels in fetuses. This is also the result of worsening intrauterine environment in fetuses (33). In this study, we found that among children with hypospadias, those with SGA had significantly shorter ASD, AGD-2, and AGD-1 than those with AGA, and those with LBW had significantly shorter ASD and AGD-1. This may indicate more proximal disruption of intrauterine androgen action in SGA infants and further suggest that the occurrence of hypospadias may be related to intrauterine growth restriction caused by early placental insufficiency (34). However, some studies have shown that the association between preterm birth and hypospadias is not significant, with SGA playing an important role (2, 22, 29). Previous study showed no difference in AGD-2 and ASD between full-term and preterm boys, which coincides with our findings (29). Therefore, the specific underlying associations need further in-depth research to validate.

This study has certain limitations. As a retrospective cross-sectional study, the etiology of hypospadias is complex and diverse, making it difficult to determine. Therefore, we not only investigated the associations among different severities of hypospadias but also discussed the impact of maternal-fetal factors, such as FGR. However, the sample size of this study was relatively small, and other pregnancy factors, such as maternal exposure to endocrine disrupting chemicals, were not included. Genetic and/or environmental factors may play different roles in the occurrence of hypospadias, with different windows of action, exposure levels, or exposure types. More basic research is needed to understand the mechanisms involved. Despite these limitations, our study is a useful addition to the existing research on the etiology of hypospadias. For example, in some cases, a short AGD may be related to environmental factors during the fetal period (such as changes in hormone levels), so measuring AGD simultaneously in studies can provide deeper insights into the etiology of hypospadias and may reveal other potential developmental issues.

## Conclusion

This study revealed that compared to the control group, children with hypospadias exhibited significantly shortened AGDs, with those having proximal hypospadias showing even shorter AGDs than those with distal and middle cases. Additionally, it was observed that children with hypospadias who also had FGR demonstrated even more shortened AGDs. Further prospective cohort studies in the future may help identify specific risks and pathophysiological mechanisms.

## Data Availability

The raw data supporting the conclusions of this article will be made available by the authors, without undue reservation.
