# Supplementary figures and images for: Boys born with hypospadias and fetal growth restriction exhibit shorter anogenital distances: a retrospective cross-sectional study
Source: Front Pediatr. 2025 Jul 9;13:1602368. doi: 10.3389/fped.2025.1602368 (PMC12283591; doi:10.3389/fped.2025.1602368)

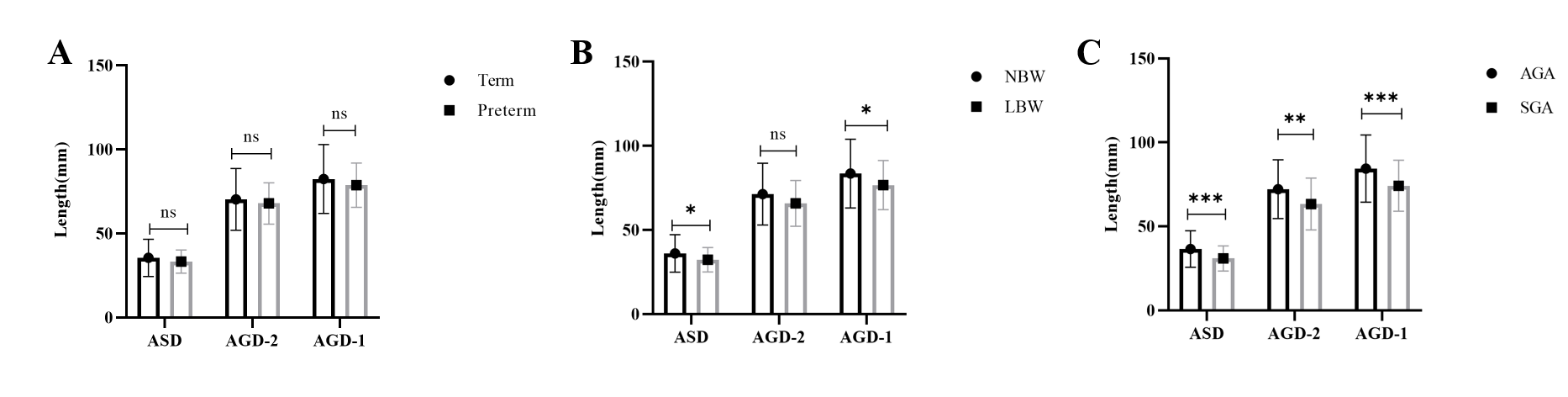

Supplement: Supplementary file 1 [file Image1.tif]
